# Prevalence and associated factors of smoking among chinese adolescents: a school-based cross-sectional study

**DOI:** 10.1186/s12889-023-15565-3

**Published:** 2023-04-11

**Authors:** Bingliang Lin, Xin Liu, Wenlong Lu, Xiaobing Wu, Yanyan Li, Ziyang Zhang, Rongyin Fu, Luge Zhang, Jingfan Xiong

**Affiliations:** 1 Shenzhen Center for Chronic Disease Control, No.2021 Buxin Road, Luohu District, Shenzhen, 518001 China; 2 Chinese Center for Disease Control and Prevention, No.27 Nanwei Road, Xicheng District, Beijing, 100032 China

**Keywords:** Adolescents, Smoking, Prevalence, Associated factors

## Abstract

**Background:**

Shenzhen has made great efforts to address the tobacco epidemic in the past decade. This study aims to evaluate the current status of the tobacco epidemic among adolescent in Shenzhen, China.

**Methods:**

The multi-stage random cluster sampling method was used in the school-based cross-sectional study in 2019 and a total of 7,423 junior and high school (both senior and vocational) students were recruited. Information on cigarette use was collected by the electronic questionnaire. Logistic regression analysis was used to examine the associations between current cigarette use and associated factors. ORs with their 95% CIs were reported.

**Results:**

The prevalence of current cigarette use among adolescents was 2.3%, with boys (3.4%) significantly higher than girls (1.0%). Smoking rates in junior high schools, senior high schools, and vocational senior high schools were 1.0%, 2.7%, and 4.1%, respectively. The results of multivariate logistic regression analysis indicated that gender, age, parental smoking, teachers smoking in schools, friends smoking, exposure to tobacco marketing, and misconceptions about cigarette use were associated factors for adolescent smoking behaviour.

**Conclusions:**

The prevalence of current smoking was relatively low among adolescent in Shenzhen, China. Personal characteristics, family, and school were associated with current adolescent smokers.

## Background

Tobacco use is one of the most serious public health problems in the world [1, 2]. Smoking was the second largest global disease burden in 2019 and is related to chronic obstructive pulmonary disease, cardiovascular disease, and cancer [2–4]. There are 1.14 billion smokers worldwide in 2019, and approximately 306 million adult smokers in China, with the largest smoking population worldwide [5, 6]. Most adult smokers initiate smoking before the age of 18, which increases their susceptibility to nicotine addiction and therefore makes it difficult to quit, leading to severe cumulative health risks and a reduced life expectancy [7–9]. The high smoking rate among adolescents in recent decades has raised public attention [10, 11].A meta-analysis among Chinese adolescents showed that the prevalence of smoking among Chinese adolescents increased rapidly from 1996 to 2010 [12]. The 2019 Global Youth Tobacco Survey (GYTS) in China showed that the adolescent smoking rate was 4.7%, and the prevalence of smoking among adolescents in different cities and regions in China vary greatly [13]. For example, in 2019, the current prevalence of tobacco among adolescents in Shandong Province was 2.1%, compared to 6.9% in Hebei Province [14, 15].

From 2012 to 2013, Shenzhen carried out the construction of smoke-free schools in the city and finally achieved full smoke-free in all schools. Meanwhile, a survey of smoking-related behaviors was conducted for secondary school students, and the prevalence of former smokers and current smokers was 12.65% and 2.83%, respectively [16]. Furthermore, in 2014, the Shenzhen government passed legislation to implement a city-wide ban on smoking in a wide range of places, including public places, workplaces, and public transportation [17]. Notably, Shenzhen revised its tobacco control regulations in 2019, which was described as one of the most stringent tobacco control regulations in China, including regulating e-cigarettes for the first time, banning the sale of tobacco products to minors, and completely prohibiting tobacco advertising, promotion, and sponsorship as core elements of tobacco control.

Evaluating smoking prevalence and examining the associated factors among adolescents is essential to develop more targeted and effective adolescent tobacco control policies and achieve smoke-free Shenzhen. Previous studies have shown that the factors influencing adolescent smoking behavior are complex and multiple such as sex, friends smoking, parental and teachers smoking etc [14, 15]. In 2021, the U.S. National Youth Tobacco Survey (NYTS) showed that adolescent current smoking rate was 1.5%, and found that tobacco accessibility contributed to tobacco use among adolescent [18]. In addition, a tobacco survey from Hong Kong in 2017 for adolescents showed that the prevalence of tobacco among adolescents was 2.5%, and concluded that economic inequality, misconceptions about the dangers of tobacco and positive attitudes towards smoking behaviors play a positive role in adolescent smoking [19].

About 8.7 million deaths worldwide in 2019 were linked to tobacco use, with nearly a third of these occurring in China, and without effective tobacco control measures, tobacco will cause about 3 million deaths per year in China after 2050 [2, 20]. It is of concern that approximately 100 million of the 300 million Chinese smokers currently under the age of 30 will eventually die as a result of tobacco [21]. In addition to health, smoking causes huge socio-economic costs in China such as medical costs, lost productivity and tobacco-related poverty. In 2014, the total economic cost of tobacco use in China was $57 billion, including the cost of treating tobacco-related diseases, the economic loss from reduced productivity and labour force, and the loss of millions of Chinese families to poverty due to tobacco-related diseases and premature deaths [22].

Although some research regarding adult smoking has been conducted in Shenzhen, studies on the prevalence of adolescent smoking and associated factors are still lacking [23–25]. Hence, this study is the first to evaluate the prevalence of smoking among adolescent students in Shenzhen after the implementation of smoke-free legislation and explore the associated factors of adolescent smoking.

## Methods

**Fig. 1:**
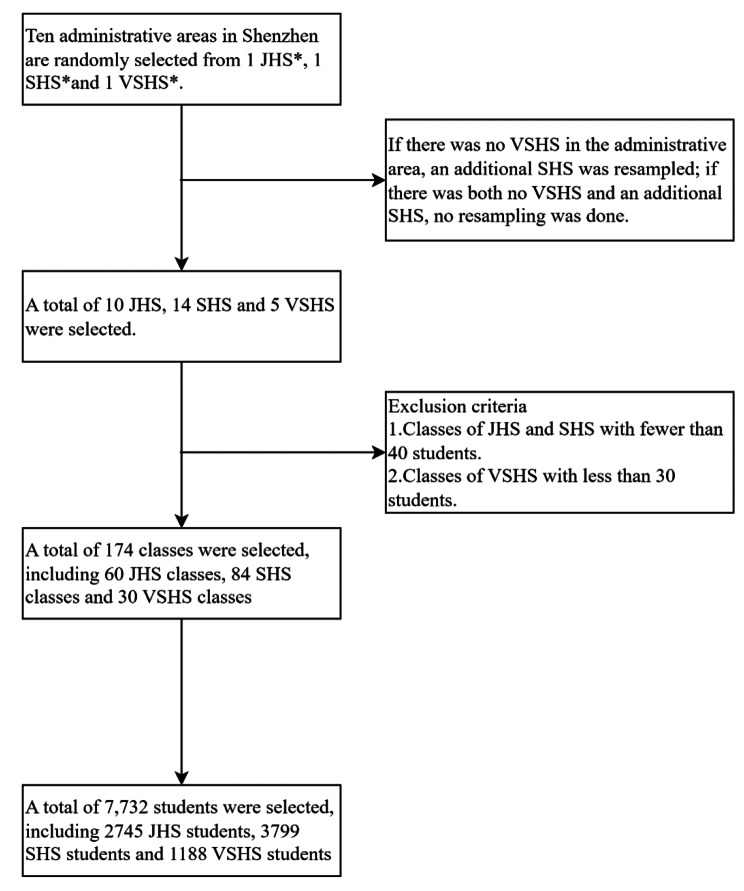
Sampling process chart JHS: Junior high school SHS: Senior high school VSHS: Vocational senior high school

ParticipantsThe present school-based cross-sectional study was conducted in Shenzhen in 2019. Participants are students from junior high school (JHS), senior high school (SHS), and vocational senior high school (VSHS) recruited from 10 administrative districts in Shenzhen by using the multi-stage random cluster sampling method. The ten administrative districts of Shenzhen are Luohu, Futian, Nanshan, Baoan, Longgang, Longhua, Guangming, Dapeng, Yantian and Pingshan. The schools used for the sampling include all public and private schools with junior and high school grades. In the first stage of sampling, Probability Proportionate to Size method Sampling (PPS) was used in each administrative district to select one JHS, one SHS, and one VSHS. If there was no VSHS in the administrative district, we choose another SHS instead. In particular, we have selected only 1 JHS and 1 SHS in the district as there is no VSHS and another SHS in Dapeng District. In the second stage, 2 classes were randomly selected from each grade of each school, for a total sample of 174 classes at all levels of school type (60 JHS classes, 84 SHS classes and 30 VSHS classes), and all students in the selected classes were eligible to participate. Classes of JHS and SHS with fewer than 40 students, and of VSHS fewer than 30 students were excluded from the sampling frame. All stages of sampling were completed by the Tobacco Control Office of the China Center for Disease Control and Prevention. The details were showed in Fig. 1.

### Data collection

A self-administered questionnaire, developed based on the Chinese version of the GYTS, was used to collect information on primary information (school, grade, class, and individuals), tobacco use, cessation, e-cigarette use, second-hand smoke exposure, tobacco advertisements and promotions, smoking perceptions and attitudes, and anti-tobacco information. The questionnaire was reviewed, checked, approved by experts, and revised after a pilot study. Before the survey, the investigators communicated with the schools in advance and determined the investigation time. Before the survey, we sent written letters to parents to obtain their consent for this study. On the day of the survey, participants were organized to go to multimedia classrooms provided by schools and use computers to complete the questionnaire. In particular, to ensure the accuracy of the study results, all students completed the questionnaire independently with no teachers present. All researchers were strictly trained to protect students’ privacy and ensure the confidentiality of personal data. Additionally, ethics approval (reference number: No. SZCCC-2019-028-01) was obtained from the Ethical Review Committee of the Shenzhen Center for Chronic Disease Control, and all steps and methods in the investigation process were carried out in accordance with the documents approved by the Committee. Informed consent was obtained in writing from the parents or guardians of all participants for this survey.

### Measurements

The smoking behaviours of the participants were evaluated in the questionnaire by the following items: (1) Have you ever tried or experimented with cigarette smoking, even one or two puffs? (yes, no); (2) During the past 30 days, on how many days did you smoke cigarettes? (0, 1–2, 3–5, 6–9, 10–19, 20–29, 30 days). Ever smokers were identified if participants answered that they had ever tried more than one puff of cigarettes. Current smokers were identified if participants answered that they had smoked at least 1 day during the past 30 days.

Explanatory variables that are taken into consideration include age(≤ 13, 14 ~ 16, > 16 years), gender (girls, boys), school level (JHS, SHS and VSHS), parental education level (< middle school graduate, middle school graduate, >middle school graduate), family economic status (average, wealth), whether have only one child (yes, no), whether studying in boarding in school (yes, no), a number of parent smokers (no, one parent, both), frequency of noticing teachers’ smoking in schools (never, sometimes, almost every day, indifferent), friends smoking (yes, no), ever exposure to tobacco product marketing in last 30 days(yes, no), have received health education on the dangers of tobacco in class (yes, no, indifferent), considered smoking more attractive (yes, no, indifferent) and perceived comfortable smoking in social occasions (yes, no, indifferent).

### Statistical analysis

Descriptive analysis was performed to estimate the prevalence of current and ever-smokers among middle and high school students. To explore the associated factors of adolescent smoking, univariate logistic regression analyses were performed to preliminarily determine the potential factors (in Tables 1 and 2) that are associated with current smoking among adolescents. Baseline variables that were considered to be associated with adolescent smoking or that showed a univariate relationship with the outcome were entered into a multivariate logistic regression model. Given the number of events available, the variables included were carefully selected to ensure parsimony of the final model. Additionally, likelihood ratio test was applied to evaluate probability of models with less predictors compared with the full model (all p < 0.05). The results proved that the full model could fit better in the dataset. The effect values were reported by odd ratios (OR) with a 95% confidence interval (95%CI). All analyzes were performed using R 3.6.1 software.

**Table 1:** Univariate analysis of the associations between demographic characteristics and the risk of adolescent current smoking

Variables	Total(*N* = 7423)	Smokers (*N* = 170, %)	*OR*	*95%CI*	*P* value
Gender					
Girls	3490	35(1.0)	1.000		
Boys	3933	135(3.4)	3.509	2.442–5.178	< 0.001
Age					
≤ 13	1447	10(0.7)	1.000		
14–16	4070	71(1.7)	2.551	1.312–4.960	0.006
> 16	1906	89(4.7)	7.039	3.647–13.582	< 0.001
School level					
JHS^a^	2663	27(1.0)	1.000		
SHS^b^	3629	97(2.7)	2.681	1.772–4.197	< 0.001
VSHS^c^	1131	46(4.1)	4.139	2.579–6.772	< 0.001
Father’s education level					
<Middle school graduate	466	18(3.9)	1.000		
Middle school graduate	1788	43(2.4)	0.613	0.356–1.099	0.087
>Middle school graduate	5169	109(2.1)	0.536	0.331–0.920	0.016
Mother’s education level					
<Middle school graduate	840	21(2.5)	1.000		
Middle school graduate	1989	40(2.0)	0.800	0.474–1.390	0.414
>Middle school graduate	4594	109(2.4)	0.947	0.489–1.347	0.824
Family economic status					
Average	3452	76(2.2)	1.000		
Rich	3971	94(2.4)	1.077	0.794–1.465	0.634
Only child					
No	5291	118(2.2)	1.000		
Yes	2132	52(2.4)	1.096	0.782–1.516	0.586
Boarding in school					
No	3726	73(2.0)	1.000		
Yes	3697	97(2.6)	1.348	0.993–1.838	0.057

JHS^a^: Junior high school

SHS^b^: Senior high school

VSHS^c^: Vocational senior high school

**Table 2:** Univariate analysis of the associations between related factors and the risk of adolescent current smoking

Variables	Total(*N* = 7423)	Smokers (*N* = 170, %)	*OR*	*95%CI*	*P* value
Parents smoking					
No	3999	64(1.6)	1.000		
One parent	3205	84(2.6)	1.655	1.193–2.306	0.003
Both	82	11(13.4)	9.526	4.591–18.169	< 0.001
Unknown	137	11(8.0)	5.368	2.725–10.030	< 0.001
Frequency of teachers smoking in schools					
Never	3912	36(0.9)	1.000		
sometimes	1792	65(3.6)	4.052	2.703–6.170	< 0.001
Almost every day	444	50(11.3)	13.663	8.822–21.367	< 0.001
Unknown	1275	19(1.5)	1.629	0.913–2.815	0.0874
Friends smoking					
No	5005	14(0.3)	1.000		
Yes	2418	156(6.5)	24.586	14.739–44.572	< 0.001
Exposure to tobacco product marketing					
No	5752	69(1.2)	1.000		
Yes	1671	101(6.0)	5.298	3.890–7.257	< 0.001
Have received health education on the dangers of tobacco in class					
No	4255	76(1.8)	1.000		
Yes	2054	66(3.2)	1.825	1.305–2.548	< 0.001
Unknown	1114	28(2.5)	1.417	0.900-2.171	0.119
Considered smoking more attractive					
Indifferent	2371	79(3.3)	1.000		
Yes	413	49(11.9)	3.906	2.675–5.652	< 0.001
No	4639	42(0.9)	0.265	0.180–0.384	< 0.001
Perceived comfortable smoking in social occasions					
Indifferent	863	53(6.1)	1.000		
Yes	286	73(25.5)	5.238	3.574–7.724	< 0.001
No	6274	44(0.7)	0.108	0.072–0.162	< 0.001

## Results

### Demographic characteristics

Among the 7,732 completed questionnaires, 309 samples with logical errors were excluded from the analysis, and the response rate of the questionnaire was 96.0%. In total, this study included 7423 students recruited from 10 administrative districts in Shenzhen, China. The median age of the sample was 15 years. There were 3,490 (52.98%) girls and 3,933 (47.02%) boys, and 2,263 (35.87%) were JHS students, 3,629 (48.89%) were in SHS students and 1,131(15.24%) were VSHS students.

### Associated factors for current smoking among adolescents

In this study, 2.3% of students reported they were current smokers (CS) (Table 1), with the largest number being boys (3.4%) and then girls (1.0%). The prevalence rate of CS varied by age, with 4.7%, 1.7%, and 0.7% in older than 16 years old, 14–16 years, and in the younger than or equal to 13 years age group, respectively. Among them, we observed that the current smoking rate was highest in VSHS students (4.1%), and then SHS (2.7%), and lowest in JHS students (1.0%). Those students who had fathers with a high school education or higher (2.1%) had lower current smoking rate than those with fathers below a high school education (3.9%). Students whose parents smoked were more likely to be CS than those with neither parent smoking, 13.4% versus 1.6%. In addition, 11.3% and 3.6% of high school students with current smoking status indicated that they saw their teachers smoking on campus almost every day or sometimes, while only 0.9% among those who had never seen them. Those students with smoking friends had higher current smoking rates than those without such friends, 6.5% vs. 0.3%. Approximately 6.0% of CS reported that they had been exposed to tobacco product marketing, and 1.2% of them reported they had not. Furthermore, more students were educated about the dangers of tobacco than those who were not, 3.2% vs. 1.8%. Regarding the relationship between smoking and personal attractiveness, 11.9% of CS believe that smoking makes people more attractive, 0.9% hold the opposite view, and 3.3% believe that there is no relationship. Among current smoking students, 25.5% believe that smoking in social settings makes people feel more comfortable, but 0.7% disagree with this idea and 6.1% think that it makes no difference. The details were showed in Table 2.

The factors with statistically significant differences in Tables 1 and 2 were selected and multivariate logistic regression models were used to explore associated factors of currently smoking adolescents. As shown in Table 3, gender, age, parents smoking, frequency of teachers smoking in schools, friends smoking, exposure to tobacco product marketing, considered smoking more attractive, and perceived comfortable smoking at social occasions were significant independent factors of current smoking adolescents. Compared to girls, the OR of boys was 1.557 (95% CI: 1.004–2.415). Older than 16 years old (OR = 2.623, 95%CI = 1.205–5.709), friends smoking (OR = 10.991, 95%CI:6.126–19.717), exposed to tobacco marketing (OR = 4.352, 95%CI:2.996–6.321), consider smoking more attractive (OR = 2.094, 95%CI:1.266–3.464) and almost every day teachers are seen smoking at school (OR = 2.893, 95%CI:1.669–5.013) were also strongly independent factors of smoking currently adolescents. Regarding parents smoking, the OR was 1.634 (95% CI 1.099–2.428) for adolescents with one parent smoking, 5.348 (95% CI 2.092–3.672) for both parents smoking, and 4.078 (1.834–9.066) for those without knowledge of parental smoking status. Additionally, the OR of current smokers for perceived more comfortable smoking in social situations was 4.140 (95%CI:2.583–6.633), and those who felt uncomfortable was 0.201 (95%CI:0.127–0.317).

**Table 3:** Multivariate analysis of the associations between related factors and the risk of adolescent current smoking

Variables	*B*	*SE*	*OR (95% CI)*	*P* value
Gender				
Girls			1.000	
Boys	0.443	0.224	1.557(1.004–2.415)	0.048
Age				
≤ 13			1.000	
14–16	0.706	0.394	2.026(0.936–4.385)	0.073
> 16	0.964	0.397	2.623(1.205–5.709)	0.015
Parents smoking				
No			1.000	
One parent	0.491	0.202	1.634(1.099–2.428)	0.015
Both	1.677	0.479	5.348(2.092–13.672)	< 0.001
Unknown	1.406	0.408	4.078(1.834–9.066)	0.001
Frequency of teachers smoking in schools				
Never			1.000	
sometimes	0.399	0.240	1.490(0.932–2.385)	0.096
Almost every day	1.062	0.281	2.893(1.669–5.013)	< 0.001
Unknown	-0.004	0.317	0.996(0.535–1.853)	0.990
Friends smoking				
No			1.000	
Yes	2.397	0.298	10.991(6.126–19.717)	< 0.001
Exposed to tobacco marketing				
No			1.000	
Yes	1.466	0.190	4.352(2.996–6.321)	< 0.001
Considered smoking more attractive				
No difference			1.000	
Yes	0.739	0.257	2.094(1.266–3.464)	0.004
No	-0.298	0.226	0.742(0.476–1.156)	0.187
Perceived comfortable smoking in social occasions				
No difference			1.000	
Yes	1.421	0.241	4.140(2.583–6.633)	< 0.001
No	-1.605	0.233	0.201(0.127–0.317)	< 0.001

## Discussion

This is the first article published on the status of youth smoking since the implementation of smoke-free legislation in Shenzhen. In the past, Shenzhen’s tobacco control efforts were primarily aimed at addressing the adult tobacco epidemic, on the basis of which numerous studies have been conducted [17, 23]. Developing sustained and well-established monitoring strategies can contribute to creating effective policies and measures for adolescents that can significantly reduce adolescent smoking rates.

According to our study, the overall current smoking rate among adolescents was 2.3% in Shenzhen, with boys (3.4%) significantly higher than girls (1.0%). There was a positive correlation between adolescent current smoking and their age, with the lowest rates in groups younger than or equal to age 13 (0.7%) and the highest rates in groups older than age 16 (4.7%). The current smoking rate also varies greatly by school type, with the highest in VSHS at 4.1%, the lowest in JSH at 1.0%, and SHS at 2.7%. The overall current smoking rate was virtually unchanged from the 2012 Shenzhen Youth Tobacco Survey, 2.8% compared to 2.3% [16]. Compared with the 2019 GYTS China survey, the overall prevalence of the current smoking rate of adolescents in 2019 was significantly decreased, (2.3% vs. 5.9%), with similar patterns in boys currently smoking rates (3.4% vs. 9.6%), but little change in current smoking rates among girls (1.0% vs. 1.9%) [13]. This indicates the need to develop gender-specific tobacco control strategies to prevent female adolescents from smoke initiation, and to assist cessation of smoking female adolescents in the future. And then, our results showed that current smoking rates were both lower in JHS, SHS, and VSHS than in the China GYTS 2019 survey, 1.0% vs. 3.9%, 2.7% vs. 5.6% and 4.1% vs. 14.7%, respectively [13]. Moreover, the current smoking rate is lower than in other studies conducted in countries and cities in China. For example, the 2021 National Youth Tobacco Survey (NYTS) showed that 1.5% of boys and 1.5% of girls smoked in the last 30 days, and a cross-sectional survey conducted by Lin zhu et al. suggested that the current smoking rate among adolescents was 4.26% in Fujian Province in 2019 [18, 26]. This may be related to the implementation of Shenzhen’s 2019 comprehensive smoking control regulations, which is considered to be one of the strictest related laws in China.

In this study, we determined the associated factors of current adolescent smoking. Adolescents who were boys or older than aged 16 years were more likely to present smoking status, which is consistent with the findings of Meng Wang et al. for middle and high school students in Zhejiang Province in 2012 [27]. Additionally, misconceptions about smoking were positively associated with the risk of smoking among adolescents [28, 29]. Adolescents who perceived smoking as more attractive (OR = 2.094, 95%CI:1.266–3.464) and comfortable in social environments (OR = 4.140, 95%CI:2.583–6.633) were at higher risk of using cigarettes and easier to accept e-cigarettes or other tobacco products [30, 31]. In particular, parents, teachers, and peers contribute strongly to adolescent smoking [32]. Parental smoking behaviour and tolerant attitudes towards tobacco use are critical to initiating and maintaining smoking among adolescents [33, 34]. Either one (OR = 1.634, 95%CI:1.099–2.428) or both parents smoked (OR = 5.348, 95%CI:2.092–13.672) made adolescents more likely to continue smoking. We did not further analyze the respective roles of fathers and mothers on adolescent smoking, but previous studies indicated that adolescents were more likely to smoke when their mothers smoked [35] and that maternal smoking had a greater effect on girls than boys [35, 36]. In addition, parental divorce or separation, and living with other family members who smoked but not parents were all more likely to lead to adolescent smoking [27, 35]. Notably, a survey of 33,408 adolescents showed that current smoking was strongly negatively associated with maternal care and control, but positively associated with paternal control [37]. In this context, the traditional Chinese parenting style (strict father and loving mother) may lead to rebellious behavior in adolescents, such as smoking. Therefore, a comprehensive review of the combined effects of family on adolescent smoking behavior is necessary in the future.

Similar to previous studies [38, 39], adolescents surrounded by smoking friends were more likely to smoke at present [38, 39]. Our study also showed that the association between current student smoking and teacher smoking depended on the extent to which teachers’ smoking behaviour was seen. There was no relationship between students smoking and occasionally seeing teachers smoking on campus; this risk was only significant when students noticed teachers smoking almost daily or daily. Teachers can play an essential role in shaping the behaviour of young people, as they spend most of their time at school, so Shenzhen implemented a city-wide smoke-free school strategy in 2013, which prohibited teachers from smoking in schools [40]. However, smoking teachers undermines or diminishes the authority of smoke-free regulations. Students observing teachers’ smoking behaviour may believe no restrictions exist or that existing regulations are more lenient, eventually inducing and sustaining smoking. A longitudinal study from the USA showed that tobacco advertising and promotion converts non-smoking adolescents into regular smokers and the more intense the tobacco marketing, the more likely adolescents are to smoke [41, 42]. Despite a complete ban on the promotion and sponsorship of tobacco advertising and the sale of tobacco products through vending devices in Shenzhen in 2019, we still found this link in our research. Therefore, there is a need to strengthen the enforcement of this regulation in the future to ensure that young people are protected from tobacco industry marketing.

The most notable strength of this study is its focus on the specific group of adolescents, including the description of tobacco use among adolescents and associated factors, which will help the government and health authorities to tailor anti-tobacco policies and measures for adolescents. However, this study also has some limitations. First, the cross-sectional study cannot provide causality, and further longitudinal research is needed. Second, the current smoking status among adolescents was a self-reported result without biological validation. Meanwhile, adolescent smoking is often perceived as a “bad boy” due to socio-cultural norms, so that the adolescent tended to report not smoking, which may result in underestimating the prevalence of smoking.

This study provides valuable insights into adolescent smoking behaviour and associated factors. Perennial and comprehensive surveillance strategies are important for understanding the smoking status and smoking-related factors among adolescent, which is the key to addressing youth smoking. future tobacco control policies should be more specific and customized, taking into consideration gender and smoking-related factors to provide effective cessation services. Additionally, this study demonstrates that the existing tobacco control strategies and measures in Shenzhen are insufficiently effective in discouraging female students from smoking. Future qualitative interviews with female adolescents may be needed to understand the particularities of this group in the area of tobacco control to implement effective and tailored measures. Targeting parents who smoke and developing school-based cessation or prevention programs in the context of smoke-free schools to decrease the number of adolescent smokers in schools, thus significantly reducing the number of smokers based on peer interaction, could be one of the effective ways to address the adolescent tobacco epidemic in the future.

## Conclusions

Smoking prevalence was relatively low among Chinese adolescents in Shenzhen. Personal characteristics contributed to the influence of smoking behaviour among adolescents. In addition, environmental factors related to tobacco exposure, including family, school, and tobacco marketing, were also associated with adolescent smoking behaviour. Developing a family-school-community trinity model for tobacco control, and conducting school-based anti-tobacco information and cessation interventions to reduce adolescent smoking rates, especially those with the above associated factors.

## Data Availability

All data relevant to the study are included in the article or uploaded as online supplemental information.

## Abbreviations

GYTS: Global Youth Tobacco Survey
JHS: Junior high school
SHS: Senior high school
VSHS: Vocational senior high school
PPS: Proportionate to Size Sampling
CS: Current smokers
OR: Odds ratios
CI: Confidence intervals
NYTS: National Youth Tobacco Survey
